# Molecular Interplay Between COVID‐19 and Stroke: Experimental Insights Into MicroRNA‐Regulated Neuroinflammation

**DOI:** 10.1155/cjid/5657680

**Published:** 2026-09-17

**Authors:** Saber Soltani, Milad Zandi, Habibollah Mirzaei, Saeedeh Ebrahimi, Hamid reza Zaheri, Samaneh Abbasi

**Affiliations:** ^1^ Department of Medical Laboratory Sciences, School of Allied Medical Sciences, Tehran University of Medical Sciences, Tehran, Iran, tums.ac.ir; ^2^ Zoonosis Research Center, Tehran University of Medical Sciences, Tehran, Iran, tums.ac.ir; ^3^ Iranian Society for Virology, Deputy for Education, Ministry of Health and Medical Education, Tehran, Iran, behdasht.gov.ir; ^4^ Department of Virology, Hepatitis Research Center, Faculty of Medicine, Lorestan University of Medical Sciences, Khorramabad, Iran, lums.ac.ir; ^5^ Department of Internal Medicine, Afzalipour School of Medicine, Kerman University of Medical Sciences, Kerman, Iran, kmu.ac.ir; ^6^ Faculty of Medicine, Abadan University of Medical Sciences, Abadan, Iran, abadanums.ac.ir; ^7^ Department of Microbiology, School of Medicine, Abadan University of Medical Sciences, Abadan, Iran, abadanums.ac.ir

**Keywords:** COVID-19, miRNAs, neurological manifestations, SARS-CoV-2, stroke

## Abstract

**Background:**

The recent COVID‐19 pandemic has resulted in a wide spectrum of neurological complications, including stroke, and emerging evidence suggests a critical role of microRNAs in their development. This study aimed to evaluate the expression levels of miR‐124, miR‐636, and miR‐485‐5p in COVID‐19 patients with and without stroke, compared with healthy individuals, to investigate their potential roles in COVID‐19‐associated neurological manifestations.

**Methods:**

This cross‐sectional study included 100 individuals: 25 COVID‐19 patients with stroke, 25 COVID‐19 patients without stroke, and 50 healthy controls. COVID‐19 infection was confirmed using RT‐PCR. Following plasma isolation and RNA extraction, quantitative real‐time PCR was performed to determine the expression levels of miR‐124, miR‐636, and miR‐485‐5p. The Kruskal–Wallis test followed by Dunn’s post hoc test was used for statistical analysis.

**Results:**

The miR‐124 expression was significantly increased in the stroke‐positive group (5.13 ± 2.48) compared to both the stroke‐negative (1.78 ± 1.03) and control groups (1.69 ± 0.64) (*p* = 0.0084). Significant differences were observed between the stroke‐positive and stroke‐negative groups (*p* = 0.0156), as well as between the stroke‐positive and control groups (*p* = 0.0333). Although not statistically significant, a trend toward increased levels was observed in the stroke‐positive group (*p* = 0.3594). miR‐636 showed no significant differences among the three groups (*p* = 0.9987). miR‐485‐5p showed a nonsignificant trend toward higher levels in stroke‐positive patients (2.15 ± 1.53 vs. 1.38 ± 1.01, *p* = 0.36).

**Conclusion:**

The results demonstrated a significant overexpression of miR‐124 in COVID‐19 patients with stroke, highlighting its potential role in COVID‐19‐associated neurological disorders. Further studies are required to elucidate the underlying mechanisms and assess its potential as a diagnostic and therapeutic biomarker.

## 1. Introduction

The global COVID‐19 pandemic, caused by Severe Acute Respiratory Syndrome Coronavirus 2 (SARS‐CoV‐2), has presented unprecedented challenges to public health systems worldwide [1, 2]. Although COVID‐19 is primarily considered a respiratory viral infection, emerging evidence suggests that SARS‐CoV‐2 can affect multiple organ systems, including the central nervous system (CNS), leading to neurological manifestations ranging from headache and anosmia to severe complications such as meningitis, encephalitis, and stroke [3]. Stroke, characterized by brain tissue damage resulting from vascular occlusion or hemorrhage, is a serious cerebrovascular complication associated with SARS‐CoV‐2 infection. Studies have shown that the mortality rate of COVID‐19‐associated stroke is higher than the global average for stroke‐related mortality [4]. Although the exact mechanisms underlying SARS‐CoV‐2‐induced neurological manifestations remain unclear, accumulating evidence suggests that viral‐mediated systemic inflammation, endothelial dysfunction, coagulopathy, and possible viral neuroinvasion may contribute to these complications [5, 6]. In this context, small noncoding RNA molecules, particularly microRNAs (miRNAs), play critical roles in regulating gene expression in various biological processes [7, 8]. Recent studies have demonstrated that viral pathogens can modulate host miRNA expression, thereby influencing viral replication and host immune responses [9]. Furthermore, miRNAs have been implicated in the pathogenesis of various neurological disorders [10, 11]. For example, miR‐124 has been shown to play a key role in neurological processes and to regulate various aspects of viral infections. It exerts anti‐inflammatory effects by reducing inflammatory mediators and suppressing microglial activation [12–14]. Similarly, miR‐636 and miR‐485‐5p have been investigated in the context of neurological disorders and viral infections. Where they may play important roles in inflammatory responses and cellular proliferation [15]. Given the increasing evidence on COVID‐19‐associated neurological complications and the regulatory roles of miRNAs, the present study aimed to evaluate the expression profiles of miR‐124, miR‐636, and miR‐485‐5p in COVID‐19 patients with and without stroke, compared with healthy individuals. Understanding the expression patterns of these miRNAs may provide valuable insights into the molecular mechanisms underlying COVID‐19‐associated neurological disorders and help identify potential diagnostic biomarkers and therapeutic targets.

## 2. Materials and Methods

### 2.1. Study Population and Sampling

This cross‐sectional observational study was conducted between April 2023 and February 2024. A total of 100 participants were enrolled and categorized into three groups: 50 healthy individuals (control group), 25 COVID‐19 patients without stroke symptoms (stroke‐negative group), and 25 COVID‐19 patients with clinically confirmed stroke (stroke‐positive group). COVID‐19 infection was confirmed using real‐time reverse transcription PCR (RT‐PCR) from respiratory samples. Stroke symptoms were evaluated by a neurologist. All stroke cases were confirmed using imaging techniques (CT or MRI), and the interval between COVID‐19 diagnosis and stroke onset ranged from 3 to 14 days. Healthy controls were age‐ and sex‐matched individuals without comorbidities or prior COVID‐19 infection (confirmed by negative RT‐PCR and clinical history). Demographic characteristics, comorbidities, and clinical features were recorded using standardized data collection forms. Written informed consent was obtained from all participants. The study protocol was approved by the Ethics Committee of Abadan University of Medical Sciences (No. IR.ABADANUMS.REC.1402.102).

### 2.2. SARS‐CoV‐2 Real‐Time RT‐PCR on Respiratory Samples

Nasopharyngeal swabs were collected in viral transport media and tested using a commercial RT‐PCR kit (Pishtaz Teb, Iran) according to the manufacturer’s instructions. Viral RNA was extracted using a commercial extraction kit (Beh Gene, Iran), and amplification targeted SARS‐CoV‐2‐specific genes (Viral N and RdRp).

### 2.3. Collection of Blood Samples and Plasma Isolation

Peripheral blood (5–10 mL) was collected from each participant into EDTA‐coated tubes to prevent coagulation. The blood samples were processed within 2 hours of collection to minimize RNA degradation. Samples were centrifuged to separate plasma. The clarified plasma was aliquoted and stored at −80°C until RNA extraction and downstream molecular analysis.

### 2.4. RNA Extraction and miRNA Expression Analysis

Total RNA, including miRNAs, was extracted from 200 μL plasma using the *miRNeasy Serum/Plasma Kit* (QIAGEN) with RNA spike‐in controls added to the lysis buffer. cDNA synthesis was performed using the *miRCURY LNA RT Kit* (QIAGEN), including the reverse transcription control. Expression levels of *hsa-miR-124-3p*, *hsa-miR-485-5p*, and *hsa-miR-636* were quantified using *miRCURY LNA SYBR Green PCR Assays* (QIAGEN) on a real‐time PCR system beside the U6 snRNA, and miR‐16 was used as an endogenous reference gene. Relative expression was calculated using the 2^−ΔΔCt^ method after confirming acceptable PCR efficiencies.

### 2.5. Statistical Analysis

Statistical analysis was performed using *GraphPad Prism 9* (GraphPad Software, USA). Data were expressed as mean ± standard deviation (SD). Differences in miRNA expression among the three groups (normal, stroke‐negative, and stroke‐positive) were assessed using the Kruskal–Wallis test, followed by Dunn’s post hoc test for pairwise comparisons. A *p* value less than 0.05 was considered statistically significant.

## 3. Results

### 3.1. Participant Characteristics

A total of 50 patients with laboratory‐confirmed COVID‐19 were included in the study (mean age: 63 years). Among them, 20 (40%) were female and 30 (60%) were male. The most common comorbidities were hypertension (44%), cardiovascular disease (22%), and diabetes mellitus (20%). Other underlying conditions included asthma (4%) and allergy (8%). All patients presented with fever, shortness of breath, and headache. Dry cough (56%), muscle pain (62%), and chills (18%) were also frequently reported. Diarrhea was observed in only one patient (Table 1).

**TABLE 1:** Baseline demographic and clinical characteristics of COVID‐19 patients.

Variables		*N* (*N* = 50)
Gender	Female	20
	Male	30

Blood Pressure	No	28
	Yes	22

Diabetes	No	40
	Yes	10

CVD	No	39
	Yes	11

Asthma	No	48
	Yes	2

Allergy	No	46
	Yes	4

Fever	No	0
	Yes	50

Diarrhea	No	49
	Yes	1

Shortness of breath	No	0
	Yes	50

Headache	No	0
	Yes	50

Muscle pain	No	19
	Yes	31

Dry cough	No	22
	Yes	28

Chills	No	41
	Yes	9
Mean age		63

*Note: N*: number; CVD: cardiovascular disease.

### 3.2. Differential Expression of MicroRNAs

**FIGURE 1:**
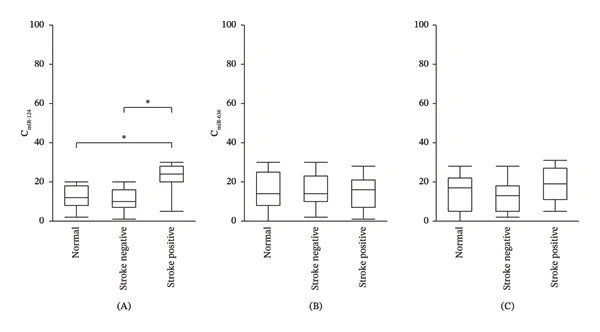
The expression levels of miR‐124 (A), miR‐636 (B), and miR‐485‐5p (C) in normal, stroke negative, and stroke positive COVID‐19 groups.

The expression levels of three microRNAs (miR‐124, miR‐636, and miR‐485‐5p) were evaluated across the study groups. Data were analyzed using the Kruskal–Wallis test, followed by Dunn’s post hoc comparisons (Table 2, Table 3, and Figure 1). The results demonstrated that miR‐124 expression was significantly elevated in the stroke‐positive group (mean ± SD: 5.13 ± 2.48) compared to both the stroke‐negative (1.78 ± 1.03) and control groups (1.69 ± 0.64) with a *p* value of 0.0084. Post hoc comparisons showed significant differences between stroke‐positive and both stroke‐negative (*p* = 0.0156) and control groups (*p* = 0.0333). Similarly, miR‐485‐5p showed a trend toward increased expression in the stroke‐positive group (2.15 ± 1.53) compared to the stroke‐negative (1.38 ± 1.01) and control groups (1.49 ± 0.98). Nonetheless, this difference did not reach statistical significance (*p* = 0.3594). In contrast, miR‐636 expression showed no significant differences among the groups (control: 2.24 ± 1.63, stroke‐negative: 2.20 ± 1.48, stroke‐positive: 2.17 ± 1.41; *p* = 0.9987).

**TABLE 2:** MicroRNAs expression level among normal, stroke negative and stroke positive COVID‐19 groups using Kruskal–Wallis test.

Gene	Normal (mean ± SD)	Stroke negative (mean ± SD)	Stroke positive (mean ± SD)	*p* value
miR‐124	1.69 ± 0.64	1.78 ± 1.03	5.13 ± 2.48	**0.0084**
miR‐636	2.24 ± 1.63	2.20 ± 1.48	2.17 ± 1.41	0.9987
miR‐485‐5p	1.49 ± 0.98	1.38 ± 1.01	2.15 ± 1.53	0.3594

*Note:* Bold *p* values indicate statistically significant differences (*p* > 0.05).

**TABLE 3:** MicroRNA expression level among normal, stroke negative, and stroke positive COVID‐19 groups using Dunn’s multiple comparisons test.

Gene	Normal (mean ± SD)	Stroke negative (mean ± SD)	Stroke positive (mean ± SD)	Summary	*p* value
miR‐124	1.69 ± 0.64	1.78 ± 1.03	5.13 ± 2.48	^∗^	(0.0333) (Normal vs. stroke positive)
				^∗^	(0.0156) (Stroke negative vs. stroke positive)

^∗^Statistically significant difference (*p* < 0.05).

## 4. Discussion

The COVID‐19 pandemic has significantly impacted global public health, extending beyond respiratory involvement to affect multiple organ systems [16]. Neurological manifestations, particularly stroke, have emerged as serious complications of COVID‐19, so that the mortality rate of COVID‐19‐caused stroke is higher than the worldwide average for stroke‐related deaths [4, 17]. Addressing the underlying molecular mechanisms of COVID‐19‐associated neurological disorders is necessary for developing appropriate diagnostic and therapeutic approaches. Current evidence suggests that COVID‐19 disrupts multiple cellular and molecular pathways [18]. Recent studies indicate that the neuropsychiatric manifestations of Long COVID have been shown to disrupt numerous cellular and molecular pathways [19]. In this context, the present study aimed to investigate the expression profiles of selected miRNAs, including miR‐124, miR‐636, and miR‐485‐5p, among 50 COVID‐19 cases (with and without stroke) and 50 healthy controls, focusing on determining their potential roles in COVID‐19‐linked neurological complications. The most important finding of this study was the significant overexpression of miR‐124 in patients with COVID‐19‐associated stroke compared to those COVID‐19 patients without stroke and healthy individuals. This overexpression was statistically significant and suggests a potential association with cerebrovascular complications in COVID‐19 patients. MiR‐124 is highly expressed in the brain, particularly in neurons, where it plays a critical role in neurogenesis, neuronal differentiation, and synaptic plasticity [20]. Moreover, miR‐124 has been shown to play anti‐inflammatory roles, commonly via inhibiting inflammatory factors and suppressing microglia function [21–24]. The mentioned overexpression of miR‐124 among COVID‐19 cases with stroke presents an intriguing paradox given its well‐established anti‐inflammatory properties. One possible explanation is that this represents a compensatory response to increased neuroinflammation. Regarding COVID‐19, systemic inflammation, endothelial impairment, and coagulopathy are known as significant factors involved in neurological disorders and stroke [25–27]. Under intense inflammatory condition and ischemia, the brain may overexpresses miR‐124 as a protective mechanism to decrease heightened inflammation and neuronal injury [28]. However, this overexpression may be insufficient to counteract the excessive inflammatory and prothrombotic state induced by SARS‐CoV‐2.

On the other hand, the increased miR‐124 level can also underscore ongoing neuronal damage [29], as investigations have reported that neuronal injury may result in the shedding of intracellular miRNAs into the circulation or changed expression status within involved tissues. In a study, Dickens et al. found that astrocytes at the site of inflammation can release microRNAs‐containing vesicles into the circulation, which in turn can increase the secretion of cytokines that stimulate peripheral immune cells to infiltrate the brain [30]. Studies have shown that viral infections can affect host miRNA expression to facilitate viral replication and modulate antiviral responses [31]. In the case of miR‐124, investigations have demonstrated that this miRNA can modulate various aspects of viral infections, such as Japanese encephalitis virus (JEV) replication via targeting dynamin2 (DNM2), a GTPase responsible for vesicle scission, as a target of miR‐124 [13]. This highlights a potential cross‐talk between miR‐124 and viral pathogens, such as SARS‐CoV‐2, where this miRNA may be involved in both host defense and disease pathology in these viral infections. Concerning miR‐485‐5p, although statistically not significant, the results of the present study suggest a trend toward overexpression in COVID‐19 patients with stroke. This miRNA has been found to be involved in various neurological and inflammatory processes [32, 33]. For example, it acts as a key regulator of cellular proliferation and inflammatory processes [15], which are involved in neuroinflammation. Although statistically not significant, the increased level underscores the necessity of further studies with larger sample sizes to investigate its exact role in COVID‐19‐induced stroke. In the present study, another evaluated miRNA, miR‐636, demonstrated no significant difference in expression among the three groups, suggesting that this miRNA may not play a significant role in COVID‐19‐associated neurological complications. Although miR‐636 has been noted for its role in inflammatory responses and cell proliferation and has been linked to neuroprotective functions and viral infections [34, 35], the absence of differential expression observed in this research suggests that, if it is involved, its participation may be different from the mechanisms associated with miR‐124 and miR‐485‐5p in strokes related to COVID‐19. The results of the present work highlight the significant role of small noncoding RNAs in COVID‐19 pathogenesis. The miR‐124 findings suggest the potential of this miRNA as a promising diagnostic biomarker for patients with COVID‐19 at higher risk for developing stroke. Moreover, miR‐124‐associated pathways can be regarded as a therapeutic candidate for reducing neuroinflammation and enhancing patient outcome in the case of COVID‐19‐induced stroke. As COVID‐19 is associated with the induction of systemic inflammation, endothelial injury, and a prothrombotic condition [36], determining how miR‐124 can affect these phenomena is critical. Recent studies have reported various miRNAs as potential factors involved in COVID‐19‐associated organ impairment and inflammation [14], underscoring the necessity of further investigation into these factors.

Several limitations should be considered when interpreting the findings of this study. First, the cross‐sectional design does not allow for causal inference between altered miRNA expression and stroke development in COVID‐19 patients; hence, longitudinal research would be needed to perform this. Second, although the sample size was sufficient to detect significant differences in miR‐124 expression, it may have limited the statistical power for identifying more subtle changes in other miRNAs, particularly miR‐485‐5p. Third, miRNA expression was assessed at a single time point, preventing evaluation of dynamic changes during disease progression and recovery. Fourth, only three candidate miRNAs were investigated, and other potentially relevant miRNAs involved in COVID‐19‐associated neurovascular pathology may have been overlooked. Finally, mechanistic and functional experiments were not performed; therefore, the biological role of miR‐124 in stroke pathogenesis remains to be confirmed by future experimental studies. Accordingly, future studies with larger sample sizes are required. Taken together, this study provides evidence of significant miR‐124 overexpression in COVID‐19 patients with stroke, highlighting its potential involvement in the neuroinflammation induced by SARS‐CoV‐2. These findings provide a foundation for future studies to further investigate the role of miR‐124 as a diagnostic biomarker and potential therapeutic target.

## Funding

No funding was received for this manuscript.

## Conflicts of Interest

The authors declare no conflicts of interest.

## Data Availability

The data that support the findings of this study are available on request from the corresponding author. The data are not publicly available due to privacy or ethical restrictions.

## References

[1] Bchetnia M. , Girard C. , Duchaine C. , and Laprise C. , The Outbreak of the Novel Severe Acute Respiratory Syndrome Coronavirus 2 (SARS-CoV-2): A Review of the Current Global Status, Journal of Infection and Public Health. (2020) 13, no. 11, 1601–1610, 10.1016/j.jiph.2020.07.011. PMC7402212 32778421

[2] Li L. , Shi X. , Wang R. et al., Cardiovascular Impact of Emerging and Re-Emerging Viruses: Pathophysiological Mechanisms, Diagnosis, and Management With a Pediatric Focus, Molecular Aspects of Medicine. (2025) 104, 10.1016/j.mam.2025.101371. 40424828

[3] Singh D. and Singh E. , An Overview of the Neurological Aspects in COVID-19 Infection, Journal of Chemical Neuroanatomy. (2022) 122, 10.1016/j.jchemneu.2022.102101. PMC9008979 35430271

[4] Pang Z. , Tang A. , He Y. et al., Neurological Complications Caused by SARS-CoV-2, Clinical Microbiology Reviews. (2024) 37, no. 4, e00131–24, 10.1128/cmr.00131-24. PMC11629622 39291997

[5] Zambrano K. , Castillo K. , Peñaherrera S. , Vasconez H. C. , Caicedo A. , and Gavilanes A. W. , Understanding Post-COVID-19: Mechanisms, Neurological Complications, Current Treatments, and Emerging Therapies, International Journal of General Medicine. (2024) 17, 6303–6321, 10.2147/ijgm.s499905. PMC11664001 39717071

[6] Ahmed W. , Feng J. , Zhang Y. , and Chen L. , SARS-CoV-2 and Brain Health: New Challenges in the Era of the Pandemic, Microorganisms. (2023) 11, no. 10, 10.3390/microorganisms11102511. PMC10609574 37894169

[7] Das K. and Rao L. V. M. , The Role of MicroRNAs in Inflammation, International Journal of Molecular Sciences. (2022) 23, no. 24, 10.3390/ijms232415479. PMC9779565 36555120

[8] Sonkoly E. and Pivarcsi A. , Advances in MicroRNAs: Implications for Immunity and Inflammatory Diseases, Journal of Cellular and Molecular Medicine. (2009) 13, no. 1, 24–38, 10.1111/j.1582-4934.2008.00534.x. PMC3823034 19175698

[9] Ojha C. R. , Rodriguez M. , Dever S. M. , Mukhopadhyay R. , and El-Hage N. , Mammalian MicroRNA: An Important Modulator of Host-Pathogen Interactions in Human Viral Infections, Journal of Biomedical Science. (2016) 23, no. 1, 10.1186/s12929-016-0292-x. PMC5081962 27784307

[10] Hussein M. and Magdy R. , Micrornas in Central Nervous System Disorders: Current Advances in Pathogenesis and Treatment the Egyptian Journal of Neurology, Psychiatry and Neurosurgery. (2021) 57, no. 1, 10.1186/s41983-021-00289-1.

[11] Plowman T. and Lagos D. , Non-Coding RNAs in COVID-19: Emerging Insights and Current Questions, Non-Coding RNA. (2021) 7, no. 3, 10.3390/ncrna7030054. PMC8482139 34564316

[12] Xu J. , Zheng Y. , Wang L. et al., miR-124: A Promising Therapeutic Target for Central Nervous System Injuries and Diseases, Cellular and Molecular Neurobiology. (2022) 42, no. 7, 2031–2053, 10.1007/s10571-021-01091-6. PMC11421642 33886036

[13] Yang S. , Pei Y. , Li X. , Zhao S. , Zhu M. , and Zhao A. , miR-124 Attenuates Japanese Encephalitis Virus Replication by Targeting DNM2, Virology Journal. (2016) 13, no. 1, 10.1186/s12985-016-0562-y. PMC4915174 27329300

[14] Rasizadeh R. , Aghbash P. S. , Nahand J. S. , Entezari-Maleki T. , and Baghi H. B. , SARS-CoV-2-associated Organs Failure and Inflammation: A Focus on the Role of Cellular and Viral Micrornas, Virology Journal. (2023) 20, no. 1, 10.1186/s12985-023-02152-6. PMC10413769 37559103

[15] Ryu I. S. , Kim D. H. , Cho H.-J. , and Ryu J.-H. , The Role of microRNA-485 in Neurodegenerative Diseases, Reviews in the Neurosciences. (2023) 34, no. 1, 49–62, 10.1515/revneuro-2022-0039. 35793556

[16] Spudich S. and Nath A. , Nervous System Consequences of COVID-19, Science. (2022) 375, no. 6578, 267–269, 10.1126/science.abm2052. 35050660

[17] Kumar D. , Jahan S. , Khan A. et al., Neurological Manifestation of SARS-CoV-2 Induced Inflammation and Possible Therapeutic Strategies Against COVID-19, Molecular Neurobiology. (2021) 58, no. 7, 3417–3434, 10.1007/s12035-021-02318-9. PMC7955900 33715108

[18] Constantinescu-Bercu A. , Lobiuc A. , Căliman-Sturdza O. A. et al., Long COVID: Molecular Mechanisms and Detection Techniques, International Journal of Molecular Sciences. (2023) 25, no. 1, 10.3390/ijms25010408. PMC10778767 38203577

[19] Caliman-Sturdza O. A. , Gheorghita R. , and Lobiuc A. , Neuropsychiatric Manifestations of Long COVID-19: a Narrative Review of Clinical Aspects and Therapeutic Approaches, Life. (2025) 15, no. 3, 10.3390/life15030439. PMC11943530 40141784

[20] Hou Q. , Ruan H. , Gilbert J. et al., Microrna miR124 is Required for the Expression of Homeostatic Synaptic Plasticity, Nature Communications. (2015) 6, no. 1, 10.1038/ncomms10045. PMC4686673 26620774

[21] Qin Z. , Wang P.-Y. , Su D.-F. , and Liu X. , miRNA-124 in Immune System and Immune Disorders, Frontiers in Immunology. (2016) 7, 10.3389/fimmu.2016.00406. PMC5047895 27757114

[22] Tazi J. , Begon-Pescia C. , Campos N. , Apolit C. , Garcel A. , and Scherrer D. , Specific and Selective Induction of miR-124 in Immune Cells by the Quinoline ABX464: A Transformative Therapy for Inflammatory Diseases, Drug Discovery Today. (2021) 26, no. 4, 1030–1039, 10.1016/j.drudis.2020.12.019. 33387693

[23] Asl E. R. , Hosseini S. E. , Tahmasebi F. , Bolandi N. , and Barati S. , MiR-124 and MiR-155 as Therapeutic Targets in Microglia-Mediated Inflammation in Multiple Sclerosis, Cellular and Molecular Neurobiology. (2025) 45, no. 1, 10.1007/s10571-025-01578-6. PMC12234947 40622635

[24] Zhao J. , He Z. , and Wang J. , MicroRNA-124: A Key Player in Microglia-Mediated Inflammation in Neurological Diseases, Frontiers in Cellular Neuroscience. (2021) 15, 10.3389/fncel.2021.771898. PMC8593194 34795564

[25] Sashindranath M. and Nandurkar H. H. , Endothelial Dysfunction in the Brain: Setting the Stage for Stroke and Other Cerebrovascular Complications of COVID-19, Stroke. (2021) 52, no. 5, 1895–1904, 10.1161/strokeaha.120.032711. PMC8078121 33794655

[26] Molaverdi G. , Kamal Z. , Safavi M. et al., Neurological Complications After COVID-19: A Narrative Review, Eneurologicalsci. (2023) 33, 10.1016/j.ensci.2023.100485. PMC10700397 38077923

[27] Marzoog B. A. , Coagulopathy and Brain Injury Pathogenesis in post-COVID-19 Syndrome, Cardiovascular and Hematological Agents in Medicinal Chemistry. (2022) 20, no. 3, 178–188, 10.2174/1871525720666220405124021. 35382728

[28] Liu X. , Feng Z. , Du L. et al., The Potential Role of microRNA-124 in Cerebral Ischemia Injury, International Journal of Molecular Sciences. (2019) 21, no. 1, 10.3390/ijms21010120. PMC6981583 31878035

[29] Wu P. , He B. , Li X. , and Zhang H. , Roles of microRNA-124 in Traumatic Brain Injury: A Comprehensive Review, Frontiers in Cellular Neuroscience. (2023) 17, 10.3389/fncel.2023.1298508. PMC10687822 38034588

[30] Dickens A. M. , Tovar-y-Romo L. B. , Yoo S.-W. et al., Astrocyte-Shed Extracellular Vesicles Regulate the Peripheral Leukocyte Response to Inflammatory Brain Lesions, Science Signaling. (2017) 10, no. 473, 10.1126/scisignal.aai7696. PMC5590230 28377412

[31] Bauer A. N. , Majumdar N. , Williams F. et al., Micrornas: Small but Key Players in Viral Infections and Immune Responses to Viral Pathogens, Biology. (2023) 12, no. 10, 10.3390/biology12101334. PMC10604607 37887044

[32] Chen X. , Zhang S. , Shi P. , Su Y. , Zhang D. , and Li N. , MiR-485-5p Promotes Neuron Survival Through Mediating Rac1/Notch2 Signaling Pathway After Cerebral Ischemia/Reperfusion, Current Neurovascular Research. (2020) 17, no. 3, 259–266, 10.2174/1567202617666200415154822. 32294039

[33] Lin X. , Mao L. , Chen Q. , Wang T. , Tao T. , and Pan L. , CircHIVEP2 Alleviates Parkinson’s Nerve Damage and Inflammatory Response by Targeting miR-485-3p, Experimental Gerontology. (2024) 188, 10.1016/j.exger.2024.112387. 38431178

[34] Bardin P. , Foussigniere T. , Rousselet N. et al., miR-636: A Newly-Identified Actor for the Regulation of Pulmonary Inflammation in Cystic Fibrosis, Frontiers in Immunology. (2019) 10, 10.3389/fimmu.2019.02643. PMC6874100 31803183

[35] Morishita A. , Yoneyama H. , Iwama H. et al., Circulating microRNA-636 is Associated with the Elimination of Hepatitis C Virus by Ombitasvir/Paritaprevir/Ritonavir, Oncotarget. (2018) 9, no. 62, 32054–32062, 10.18632/oncotarget.25889. PMC6112829 30174796

[36] Pons S. , Fodil S. , Azoulay E. , and Zafrani L. , The Vascular Endothelium: the Cornerstone of Organ Dysfunction in Severe SARS-CoV-2 Infection, Critical Care. (2020) 24, no. 1, 10.1186/s13054-020-03062-7. PMC7296907 32546188

